# Altering the Trajectory of Perfusion-Diffusion Deficits Using A BDNF Mimetic Acutely After TBI is Associated with Improved Functional Connectivity

**DOI:** 10.60124/j.pneuro.2023.10.07

**Published:** 2023-09-01

**Authors:** Gregory Smith, Pavan Thapak, Afshin Paydar, Zhe Ying, Fernando Gomez-Pinilla, Neil G. Harris

**Affiliations:** 1 Department of Neurosurgery, UCLA David Geffen School. of Medicine, Los Angeles, California, USA; 2 UCLA Brain Injury Research Center, Los Angeles, California, USA; 3 Department of Integrative Biology and Physiology, UCLA, Los Angeles, California, USA; 4 Intellectual and Developmental Disabilities Research Center, UCLA, Los Angeles, California, USA

**Keywords:** Traumatic brain injury, MRI, cerebral blood flow, mean diffusivity, BDNF, TrkB

## Abstract

Traumatic brain injury (TBI) results in metabolic deficits and functionally compromised tissue. The BDNF mimetic R13 has a significant positive effect on both tissue metabolism and behavioral outcome after TBI, indicating a promising therapeutic. To understand the mechanism of action for this intervention, we determined whether there was any association between the underlying metabolic insult and any improvement in resting state functional connectivity (FC) with MRI, or whether R13 acts through mechanisms unrelated to metabolic recovery. We found perfusion deficits could be reasonably approximated by reductions in mean diffusivity (MD) acutely after injury, because a majority of regions with low perfusion matched to regions of low MD, indicative of cell swelling. Injury alone resulted in reduced cross-brain FC and contralateral hyperconnectivity at 1d compared to sham and these were spatially coincident with regions of low MD. R13 intervention at 1–7d altered the tissue trajectory of MD pathology away from pseudo-normalization so that a greater volume of tissue remained with low MD at 7d. These same regions were associated with significant changes in cross-brain and contralateral FC in R13 treated rats compared to injured vehicle-treated rats. These data indicate a likely metabolic effect of R13 acutely after injury.

## 1. Introduction

Traumatic brain injury (TBI) is characterized by a complex pathology involving multiple molecular events across complex circuits which have been difficult to define by traditional methods. Connectivity-based analyses of the injured brain has gained prominence in the field over the last decade or so to help decipher these complexities. The approach excels on account of the whole brain, unbiased nature of the multiple data inputs used to collect information of varying types (structural diffusion data, functional magnetic resonance imaging, electroencephalography and others), and the utility of combining these datasets with behavioral or other covariates of interest to provide greater inference on the complex pathology associated with TBI. Resting state, functional magnetic resonance imaging (rsfMRI) has been particularly advantageous to the field since it provides information that reflects on not only how network level topography is affected by injury, but how specific brain regions are affected and how that impacts functionally covarying or connected brain regions.

These connectivity-based measures are superimposed upon a highly complex, dynamic pathology involving ongoing metabolic and synaptic changes among a host of other processes. We posit that to understand the structural-functional mechanism(s) that underpin changes in functional connectivity using rsfMRI, a consideration of the underlying pathology and the dynamics of its postinjury trajectory is crucial to interpreting connectivity changes after TBI. For example, the controversial interpretation of functional hyperconnectivity, or increased coupling between regions of the brain after TBI may well be resolved by determining whether the underlying tissue is initially injured but ultimately salvageable through endogenous mechanism of repair, or whether its temporal trajectory is in the direction of atrophy, which would then more likely be associated with functional and structural disconnection.

Metabolic depression is a major hallmark of TBI, especially during the acute to post-acute period after experimental TBI [1]. We have previously demonstrated the importance of early intervention by altering the cerebro-metabolic ratio to prevent subsequent axonal degeneration after contusion injury in adult rats [2]. Enhancing metabolic support through use of supplementary fuels such as pyruvate, glucose, or ketones early after injury has also shown to be beneficial [3–6]. Others have more directly shown improved outcome and reduced tissue atrophy through improvement in mitochondrial function [7]. It remains unknown whether improved outcome results from salvage of tissue previously destined to atrophy, or whether consolidation and enhancement of remaining tissue through multiple plastic mechanisms is responsible. Clearly both mechanisms would be beneficial after TBI. Altered synaptic plasticity occurs as a consequence or concurrently with trauma [8, 9], as does reduction in endogenous neurotrophic factors such as brain-derived neurotrophic factor (BDNF) which has been linked to metabolism [10, 11]. While TBI has been shown to result in both a reduction in BDNF and TrkB receptors [12], administering BDNF or overexpressing TrkB receptors after injury does not rescue behavioral deficits or prevent the loss on neurons after TBI [12, 13].

However, we have previously shown that early intervention after rat fluid percussion injury (FPI) with the small molecule, BDNF mimetic - R13 rescued both metabolic depression and counteracted reductions in synaptic proteins, while significantly improving cognitive deficits and anxiety-like behavior [14]. The effectiveness of R13, which is the prodrug of 7,8-dihydroxyflavone (7,8-DHF) and another BDNF mimetic, over direct BDNF administration is likely to be associated with its improved bioavailability; it has been shown to activate brain TrkB receptors *in vivo* [15, 16].

Given these pleiotropic effects of R13, we initiated an *in vivo* study to determine whether there was an effect on functional connectivity (FC), and whether changes in FC were coincident with spatial enhancement of tissue metabolism, or whether they were unconnected, indicating an effect more consistent with a synaptic plasticity mechanism of action through BDNF/TrkB-receptor pathways. Following the same experimental design as before [14], we acquired MRI data on cerebral blood flow (CBF), diffusion weighted imaging and rsfMRI from adult rats at 1d following FPI. We acquired the same data at 1week post-injury during which time rats received either daily R13 or vehicle. We found deficits in CBF were spatially consistent with regions of low mean diffusivity (MD) indicative of cell swelling, so that MD may be used as a global proxy for metabolic depression at least within the acute post-injury period, as supported by coincident perfusion-diffusion deficits. The major findings from the work are that altered brain FC after both injury+vehicle and improved FC in injury+R13 was primarily associated with regions of low MD, and that this spatial congruency reflected a primary metabolic effect of the drug driving FC changes.

## 2. Materials and Methods

### 2.1. Experimental Procedure

Male and female adult, sprague-dawley rats were randomly assigned to three groups: either a lateral fluid percussion injury group with daily intraperitoneal administration of either vehicle or TrkB agonist R13 (7.25 mg/kg dissolved in 5% DMSO in a 1:1 PEG300 to saline solution) from 1d-7d post-injury (n=10 and 8 respectively) or a sham anesthetic control group (n=10). MRI data were collected at 24 hrs and 7 days post-injury.

All rats were housed in pairs on a 12h light/dark cycle at 22°±2°C with *ad labitum* access to food and water. All experiments conducted were part of an UCLA animal research committee approved protocol and were performed following United States National Institutes of Health Guide for the care and use of laboratory animals.

### 2.2. TBI

Lateral fluid percussion injury was conducted using methods similar to those published by us before [3, 17, 18]. Briefly, rats were anesthetized via isoflurane vaporized in oxygen (4% induction, 1.5–2% maintenance) and received a 5 mm diameter craniectomy centered at 4.5 mm behind bregma and 2.5 mm left of the midline using a manual trephine. A plastic cap was placed into the craniotomy but leaving the intact dura, and the edges were sealed with glue (VetBond, 3M, USA) and the cap was filled with sterile saline. At approximately 30 min after anesthesia induction, the animals were connected to the lateral FPI device. Anesthesia was removed and as soon as the pedal withdrawal reflex returned in response to hindlimb toe-pinch, a mild to moderate pressure wave of 2.5±0.2 atm was directed to the intact dura. The pressure was selected to result in approximately 5–15 seconds of apnea at the time of impact.

### 2.3. MRI Acquisition

MRI data were acquired using a 7T magnet (Oxford Instruments, Carteret, NJ, USA) interfaced to a Bruker console running paravision 5.1 and BGA-12 gradients with a maximum strength of 400 mT/m (Bruker, Billerica, MA). A quadrature, receive-only surface coil (Rapid MRI International, Columbus, Ohio) actively decoupled from a whole-body birdcage coil (Bruker, Billerica, MA) used in transmit mode was used to acquire the data. Rats were placed in a custom-made acrylic cradle that allowed for isoflurane/oxygen delivery via a nose cone (0.8 l/min) and for 3-point head fixation. Once secured, rats received a 0.05 mg/kg intraperitoneal (IP) injection of dexmedetomidine immediately after which isoflurane was reduced to 0.5%. A continual 0.1 mg/kg/h subcutaneous infusion of dexmedetomidine was begun using a syringe pump (Harvard Apparatus, USA) prior to centering the brain in the isocenter of the magnet. Temperature was maintained at 37° C via homeothermic-controlled external air heating (SA11 Instruments, Inc., USA) and the breaths per minute were monitored and maintained at 40–60 bmp through fine adjustments to the isoflurane concentration.

The MRI session began with a positioning scan and field shimming followed by a brief 2-dimensional, T2 rapid acquisition with relaxation enhancement (RARE) anatomical scan (TR/TE/RARE-factor= 4216 ms/45 ms/16, respectively; 40 × 40 mm coronal field-of-view (FOV), 17 × 1 mm slices and a data matrix of 128 × 128). This was used to obtain the scanner coordinates for the coronal image slice used to obtain cerebral blood flow (CBF) using a flow-sensitive alternating inversion recovery (FAIR) standard pulse sequence using RARE readout. The coronal CBF image slice was located at 4.5mm from bregma which was centered under the craniectomy. A 3-dimensional (3D), 160 mm^3^ isometric, multi-echo gradient echo structural image was obtained with a TR of 125ms, a 20° flip angle, and 13 echoes with an effective TE range of 2.8– 52ms and a 4.08ms spacing. Resting state functional fMRI (rsfMRI) data were acquired with a FOV 96 × 96 mm^3^ and 25 × 0.75 mm coronal slices was obtained with 450 repetitions with a TR/TE of 2000/16 ms following 10 dummy scans. Dexmedetomidine infusion was discontinued and isoflurane was increased to 1–2% in oxygen during acquisition of diffusion weighted imaging (DWI) data obtained using a spin echo preparation and echo planar readout. These 3D data were acquired with 250 mm^3^ isotropic voxels with b-values of 0 and 2800 s/mm^2^, over 42 colinear, gradient vectors and using a TR/TE of 1000/26 ms. An additional 4 B0s were acquired with reverse left-right phase-encoding redundant. Following completion of the scan the animal was recovered with antisedan, an alpha-2 antagonist against dexmedetomidine (1 mg/kg, IP).

### 2.4. Data Analysis and Statistics

#### 2.4.1. CBF

A study specific template for comparison of CBF data across rats was generated using the 2D RARE data. Data were converted to nifti, brain extracted [19, 20] for input into a multivariate template construction script [21, 22]. CBF maps were generated with Bruker Paravision software, converted to nifti format and then normalized to the average CBF of the contralateral cortical grey matter. Image co-registration transformation and warp parameters obtained from generating the template were applied to the subject-level CBF data for mapping into a common space. CBF maps were normalized to the average value within the contralesional grey matter cortex and values reported as relative CBF. Brain regions with CBF deficits were defined as voxels with CBF less than 50% of contralateral grey matter.

#### 2.4.2. DWI

DWI scans were brain extracted [19, 20] then preprocessed largely using MRtrix tools [23] first by denoising and un-ringing [24], then bias field correction [24] and distortion correction using eddy correction [25–27], prior to calculating the tensor-derived indices fractional anisotropy (FA) and mean diffusivity (MD). FA maps were used to create an unbiased, common space brain template for the study [21, 22] to which all MD data were aligned by applying the affine and non-linear transformations calculated using the corresponding FA data. Deficits in MD maps were identified from all rats at a per voxel level of a z threshold of 1.7 below average sham values, where z maps were created by conducting a voxel-based comparison to sham group mean and standard deviation maps.

#### 2.4.3. Perfusion Deficits

For CBF maps, ipsilateral voxels were normalized to contralateral cortex and indicated as relative CBF (rCBF), given that contralateral changes resolve to sham levels by around 2–5hrs post-injury on this model [28, 29]. A perfusion deficit herein was operationally defined as a reduction in rCBF to 50% or more from contralateral values. This was based on prior work on this model that showed early reductions in CBF occurred to around 50% of sham levels [28–30]. Overlapping deficits in perfusion and mean diffusivity were identified in each rat by those voxels with CBF<50% of contralateral and MD<1.7z compared to sham.

#### 2.4.4. Functional Imaging

rsfMRI data were analyzed using an in-house python script that included the preprocessing steps used by us previously [31]: slice timing correction, motion correction [32–34], brain extraction [19, 20], segmentation for CSF elimination [21, 35], smoothing and band-pass filtering (0.01–0.2Hz) [32–34]. A study specific template was constructed for the RARE data using the time-averaged, brain extracted rsfMRI data as input. Individual, subject-to-template space affine transformation parameters were calculated using 12 degrees of freedom [36, 37] to enable the generation of a common brain space mask used to ensure that only brains regions shared among all rats were analyzed. The mask was applied to a rat brain atlas [38] consisting of 154 parcels that was registered to the study template resulting in 121 regions that were common to all brains. The inverse transform was calculated and applied to the masked atlas to generate subject-specific atlas templates in subject space which were then used with the processed 4D rsfMRI data to generate functional correlation matrices using Pearson correlations. The local graph network metrics: clustering coefficient and efficiency were calculated using algorithms available in brain network toolbox [39] as implemented under GraphVar [40] across the sparsity range 10–45% following Fisher z transformation of Pearson correlation data. Data were calculated for each rat and for each metric by summing the metric across brain sparsity levels: 10–45% in 5% increments.

Group level analysis of connectivity strength of Fisher z transformed data was the main outcome measure and was computed both as a difference in time within group from 1–7d between treated and untreated, or across groups at 7d. Statistics were run using a two-tailed, non-parametric permutation testing (n=5000) and a false discovery rate correction for multiple regions (P<0.01) and using apnea at the time of injury as a nuisance covariate to account for the within group variance in injury severity.

## 3. Results

FPI resulted in an average apnea time of 8.1s with a range of 5–14s immediately after injury. Injured rats were randomly assigned to receive either R13 or vehicle immediately after the first MRI session at PID (post-injury day) 1. There was no significant difference in apnea time between the two injured groups before treatment (7.6 +/− 0.75s, 8.4 +/− 0.48s, in vehicle and R13 treated injured rats, respectively).

### 3.1. Mild FPI is Associated with Significant but Transient Reductions in rCBF

**FIGURE 1:**
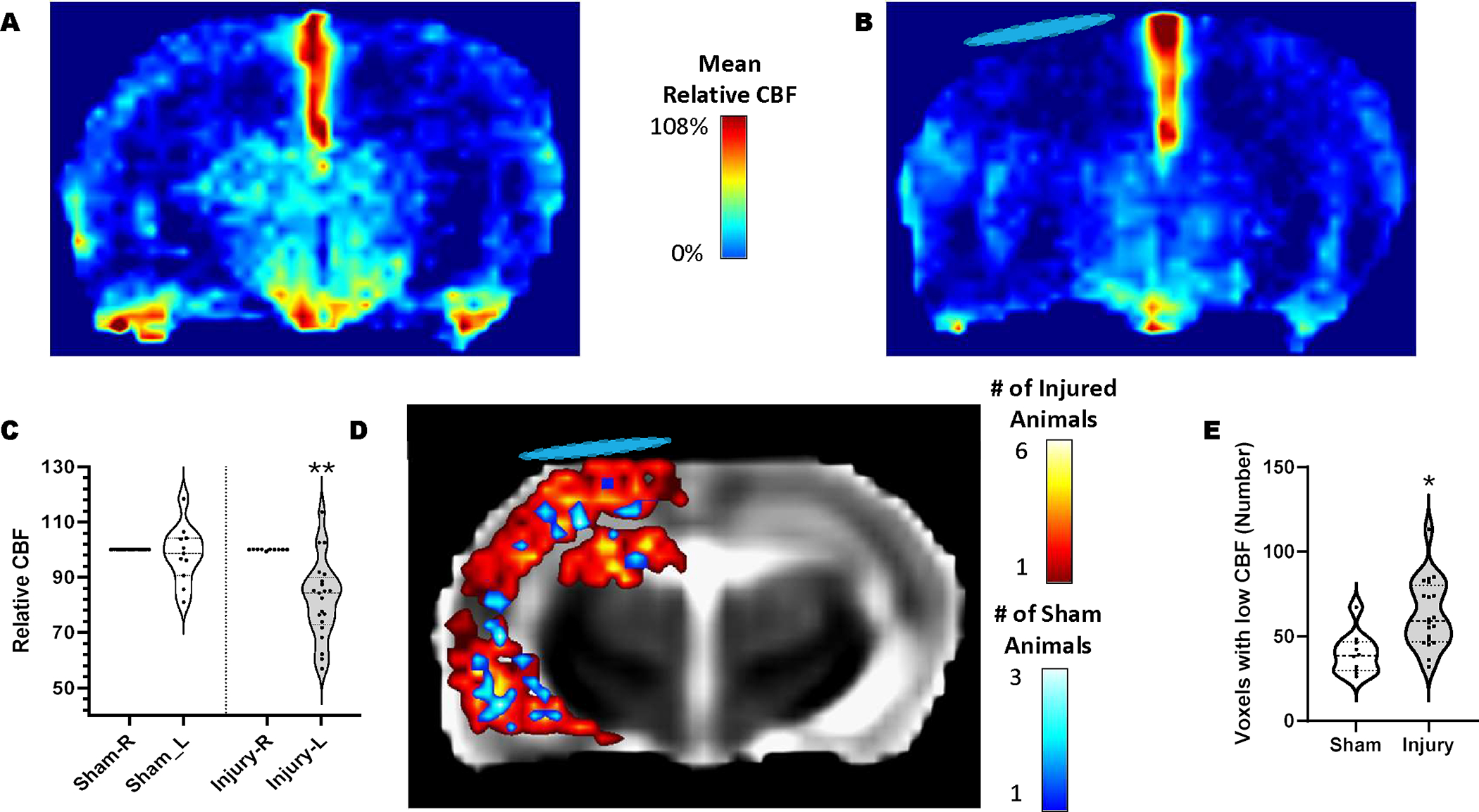
CBF is reduced ipsilaterally at 1 day PI. Mean relative CBF of **A)** sham and **B)** injured rats at PID 1. **C)** Plot of mean relative CBF in ipsilateral cortical regions after normalization to the contralateral cortex. There was no significant difference in CBF across the brain in sham rats but injured ipsilateral cortex CBF was significantly reduced when compared to sham values (** = p<0.0001). **D)** Overlap map of CBF deficits within ipsilateral cortical regions in each rat, where deficits reflect CBF less than 50% of the contralateral cortex. Pseudo-colors indicate the number of rats with CBF deficits within each brain image voxel. CBF was reduced throughout the left ipsilateral cortex in injured rats (red colors), with the greatest spatial incidence between injured rats (yellow/white colors) in brain regions directly under the craniectomy (blue disk). There were very few voxels with CBF deficits in sham rats (blue colors). **E)** Plot showing the number of ipsilateral cortical voxels with <50% CBF was increased at PID 1 compared to sham (*p<0.005). The blue disk marks the approximate location of the craniectomy.

Relative CBF (rCBF) single slice maps displayed uniform CBF across the brain in sham rats (ipsilateral cortex mean rCBF was 98% of contralateral, range 80–118%, (Figure 1A & 1C)). Injury resulted in an average 18% reduction in mean rCBF within ipsilateral cortex at PID 1 compared to shams (P<0.01; (Figure 1B & 1C)) which was on average 82% of the contralateral cortex (range of mean rCBF 57–113%). The number of individual voxels that were <50% of contralateral were significantly greater 1d after injury in both cortex and hippocampus compared to shams (P<0.05; (Figure 1D & 1E)). Importantly, there were no significant differences in the number of low CBF voxels between injured rats randomly assigned to vehicle and R13-treated groups before beginning treatment at the first MRI session (68 ± 6.9, 57± 5.2, respectively, P>0.05). By PID 7, 6 days after initiating R13 treatment, both injured groups remained with similar levels of ipsilateral rCBF, 87.6% (range 75–95%) and 87% (range 76–103%) of contralateral for vehicle and R13 groups respectively. These data were not significantly lower than in sham rats, which remained at 95.4% of contralateral (range 80–121%) indicating that CBF levels had resolved to some degree in both injured groups. Similarly, the number of voxels with rCBF <50% of contralateral was also not different between all groups.

### 3.2. Majority of Brain Regions Show a Perfusion-Diffusion Deficit at 1 Day after Injury

**FIGURE 2:**
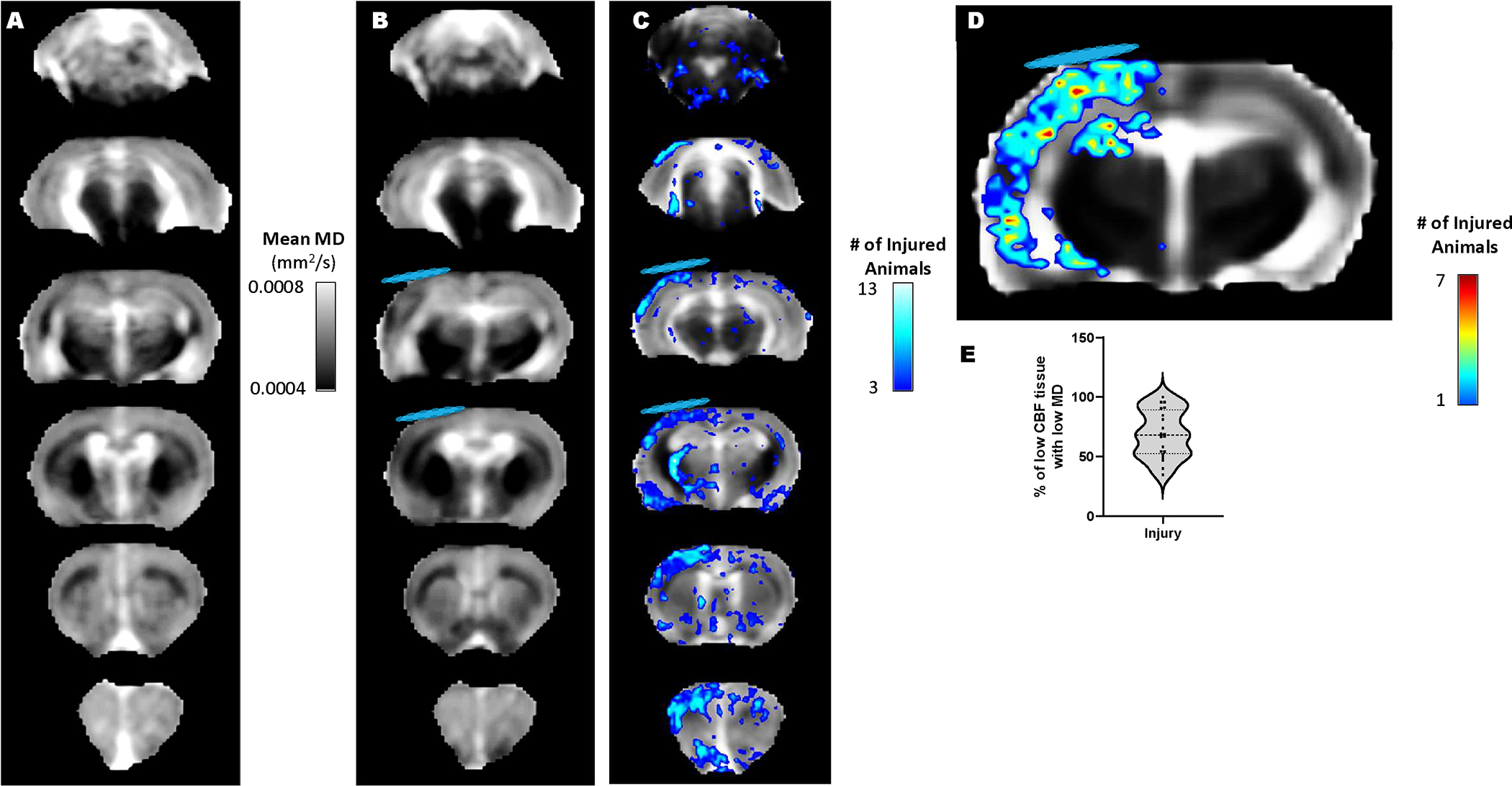
MD is reduced throughout the brain at PID 1 and is associated with CBF deficits. Whole brain voxel-based mean MD maps of **A)** the sham group and **B)** FPI injured rats. **C)** Overlap map of all injured rats showing significantly reduced MD after injury when compared to shams, identified through population-based, voxel-level z-scores (z=1.7, P<0.05). **D)** Overlap map of all injured rats at PID 1 showing the incidence of spatially congruent deficits in CBF (50% of contralateral) and low MD (z-score 1.7 less than shams). **E)** Plot showing that the CBF-MD congruence of deficits among injured rats at PID1. In injured rats the congruence was on average 68.7% with a range 34.5% to 100%. That is, 68.7% of brain regions with low CBF were associated with low MD values. Blue disk represents the approximate location of the craniectomy site.

Acute reductions in mean diffusivity (MD) after injury generally arise through cell swelling and/or reduction in the extracellular space, secondary to membrane sodium-potassium ATPase failure due to energy depletion. In order to determine whether low MD could serve as a surrogate global marker of metabolic dysfunction at PID1, we tested for an association between deficits in rCBF and low MD within the MRI image slice acquired using fair-rare MRI that was spatially co-registered to the DWI data. We first used voxel-level, population statistics of all co-registered MD data against sham controls to construct z maps to highlight regions of MD deficits within each injured rat (Figure 2A–C). In combination with the co-registered CBF data, we found that there was a 69% overlap of ipsilateral voxels (range 34–100%) between brain regions containing both CBF deficits and significantly lowered MD after injury and prior to intervention (Figure 2D & E). By 7d when mean ipsilateral rCBF had returned closer to sham levels, the low CBF / low MD overlap remained high in both injured R13 and vehicle groups (86%, range: 73–97% and 88%, range: 65–100%, respectively, P>0.05). These data indicate that at least within the first week after FPI, MD can be reasonably used as a surrogate marker of altered cerebrovascular metabolism.

### 3.3. Regions of Low MD are Associated with Altered Functional Network Architecture at PID 1

**FIGURE 3:**
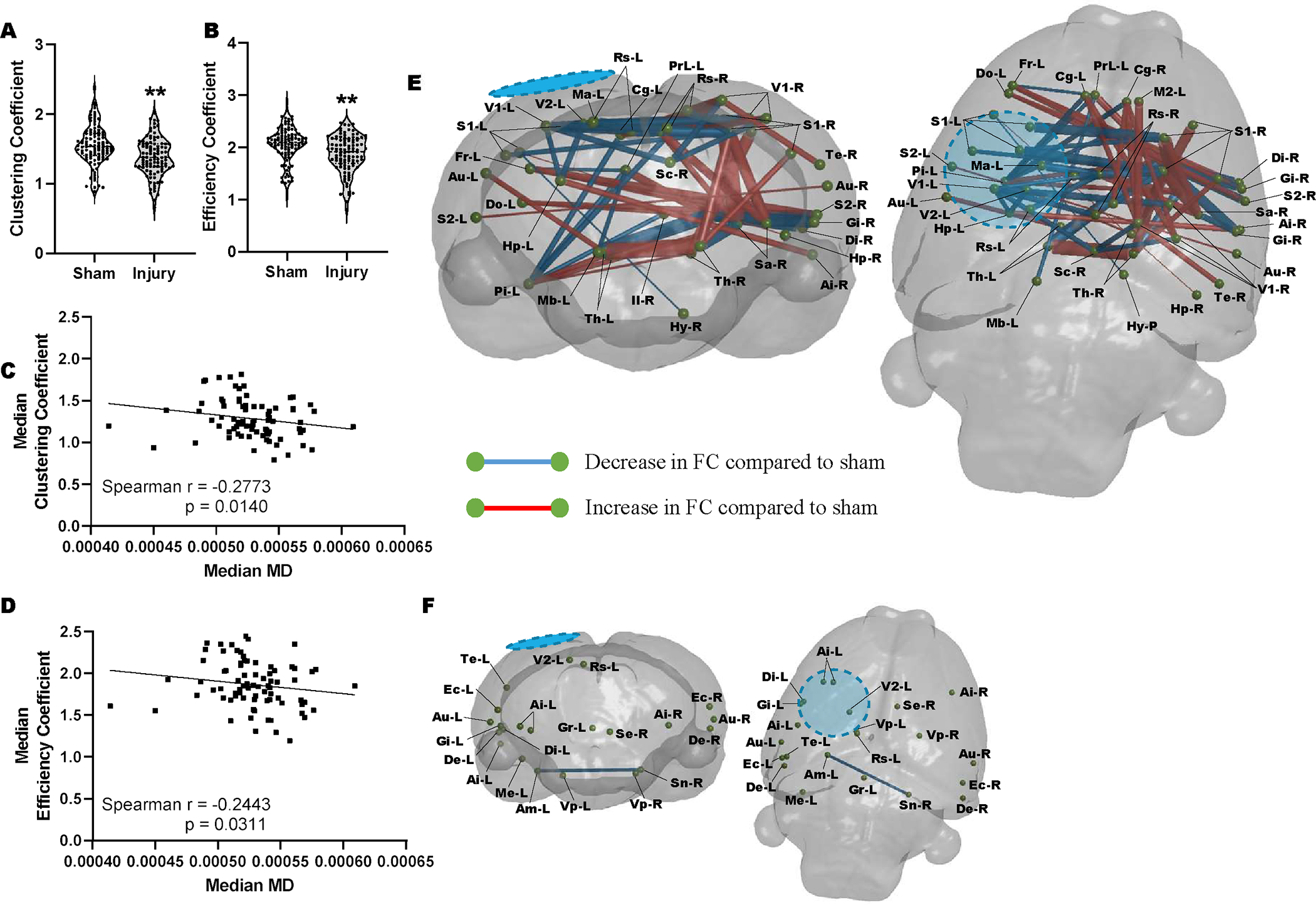
Injury-related disruptions in FC are associated with low MD at 1 day post injury. At PID 1 a significant reduction in the **A)** regional clustering coefficient and **B)** local network efficiency when pooled across all regions and compared to sham controls. There was a significant correlation between median MD across brain regions and the corresponding **C)** regional clustering coefficient (p<0.005) and **D)** local network efficiency (p<0.005). **E)** Graphical coronal and planar representation of FC showing significant alterations at PID 1 when compared to sham rats in regions of brain with low MD (p<0.01, FDR corrected, N=18–injured, 10–Sham). Significant regions of decreased FC include: sensory cortex, thalamus, striatum, hypothalamus, hippocampus. **F)** Graphical coronal and planar representation of FC showing no significant alterations at PID 1 when compared to sham rats in brain regions with MD values similar to sham controls (P<0.01, FDR corrected, N=18–injured, 10–Sham). Blue/hatched region indicate the approximate location of the craniectomy site. Key: Ai: Agranular insular cortex, Am: Amygdala, Au: Auditory cortex, Cg: Cingulate cortex, Di: Dysgranular insular cortex, De: Dorsolateral entorhinal field, Do: Dorsolateral orbital cortex, Ec: Ectorhinal cortex, Fr: Frontal cortex, Gi: Granular insular cortex, Gr: Central grey, Hp: Hippocampus, Hy: Hypothalamus, Il: Infralimbic cortex, M2: Secondary motor cortex, Ma: Medial parietal association cortex, Mb: Rest of the midbrain, Me: Medial entorhinal cortex, Pi: Piriform cortex, PrL: Prelimbic cortex, Rs: Retrospienial cortex, S1: Primary somatosensory cortex, S2: Secondary somatosensory cortex, Sa: Striatum, Sc: Superior colliculus, Se: Splenium, Sn: Substantia nigra, Te: Temporal association cortex, Th: Thalamus, V1: Primary visual cortex, V2: Secondary visual cortex, Vp: Ventral pallidum.

We next determined whether there was an association between low MD and altered functional connectivity after injury. This was led by the hypothesis that in the vehicle treated injured brain, altered functional connectivity would most likely occur within regions of metabolic depression. We interrogated rsfMRI data for the regional network properties nodal clustering coefficient (CC) and local efficiency (LE), properties that we had previously shown to be significant readouts of injury severity in the controlled cortical impact TBI model [38]. We calculated these data at the nodal (regional brain) level and averaged across a range of network sparsity levels and found that both network parameters were reduced after injury at PID 1(Figure 3A & 3B). We found that MD levels were negatively associated with both CC (r=−0.28, P<0.01) and LE (r=−0.24, P<0.05) supporting the idea that increased local connectivity occurs in regions of low MD, presumably due to disconnection of longer-range connections (Figure 3C & 3D). Additionally, regions of brain with MD values similar to sham controls median MD did not correlate with regional clustering coefficient or local network efficiency (data not shown). We tested this further by calculating edgewise FC within brain regions defined by low MD (Figure 3E) and within regions where MD was not different to sham at PID1 (Figure 3F). We found that injury-related alterations in FC that survived multiple comparisons correction were limited to regions of low MD, none survived statistical correction within brain regions containing MD not different from sham levels (P<0.01, FDR corrected, N=18–injured, 10–Sham). Within regions demarcated by low MD, injury was associated with significant reductions in FC from the ipsilateral region of primary injury i) to homotopic regions across the brain and ii) to subcortical targets, as well as iii) significant increases in FC within the contralateral hemisphere (Figure 3E).

### 3.4. R13 Intervention Altered the Trajectory of Tissue with Low MD

**FIGURE 4:**
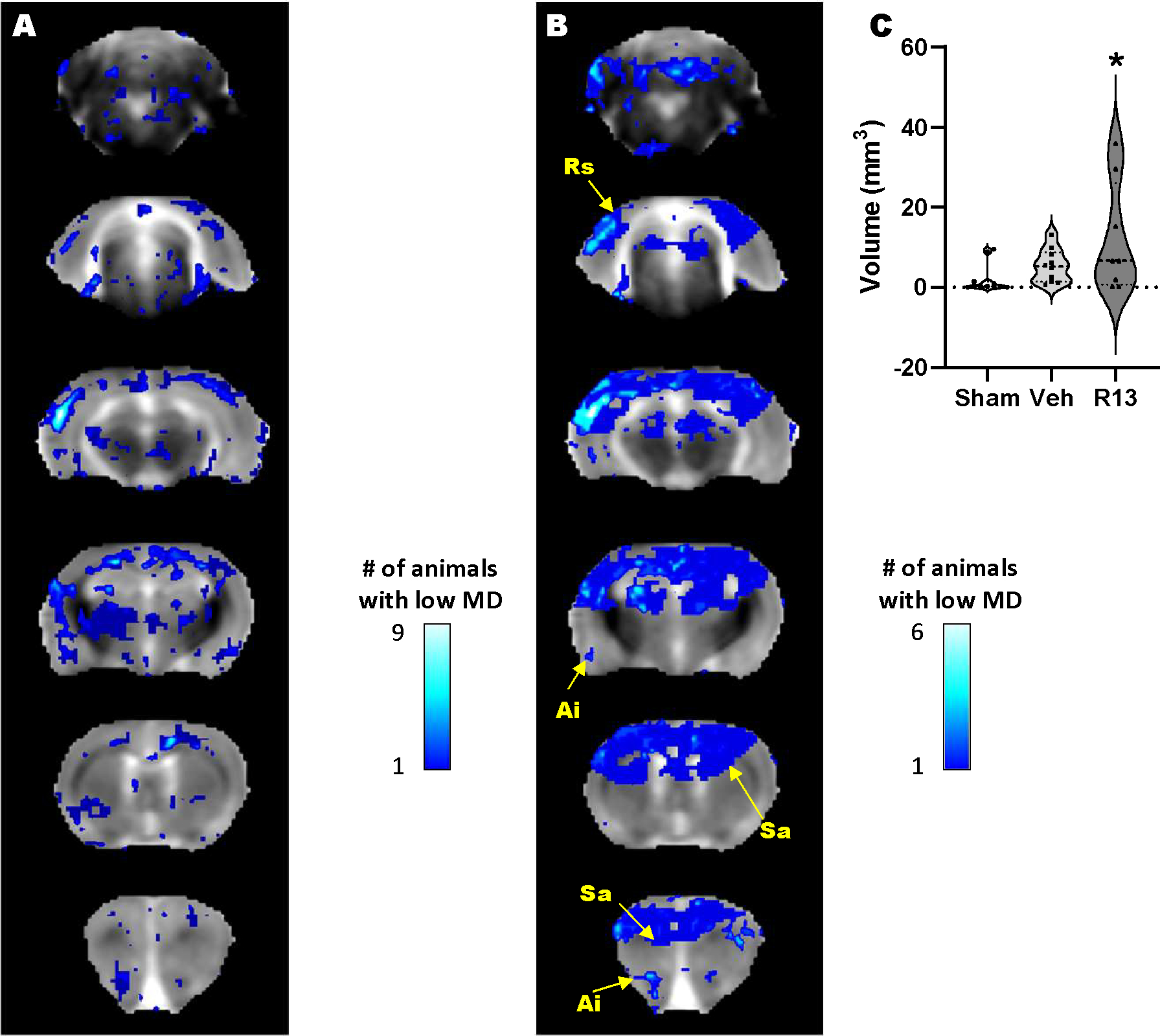
Daily R13 treatment from PID 1 results in significantly more brain tissue remaining in a low MD state at PID 7. **A)** Brain regions with enduing low MD at PID 1–7 following vehicle treatment (blue regions) include the sensory/motor cortex, the dorsal thalamus and bilateral hippocampus as well as several other cortical regions. **B)** R13 treatment resulted in a significantly greater volume of brain tissue that remained with reduced MD after treatment compared to vehicle, and include Ai: Agranular insular cortex, Rs: Retrospienial cortex, and Sa: Striatum. Location map of brain outline highlights regions with significant low MD. **C)** Plot of low MD volume indicating significantly higher volume of tissue remains in a low MD state due to R13 treatment compared to shams (*p<0.05).

The motivation for this study was also led by the likelihood that as well as a neurotrophic effect of R13 intervention, it would have a metabolic effect similar to another TrkB receptor agonist [41]. To test this, we reasoned that it would be important to determine whether R13 could improve function specifically in regions of damage that were previously associated with metabolic deficits. We first tested for a persistent metabolic effect of R13 intervention on the brain globally by examining the MD maps for regions of low MD at PID1 that remained low at PID7. The expectation here was that based on prior indications of a metabolic effect from TrkB receptor activation after TBI [41], the volume of brain regions with sustained reduction in MD at PID 7 would be lowered if daily R13 treatment from PID 1 had ameliorated cell swelling through improvements in metabolism by PID 7. Surprisingly, we found the opposite effect in the R13-treated rats, although there was a good deal of variation within group (Figure 4A & 4B). While the volume of tissue with sustained low MD in vehicle-treated injured rats was not significantly different from shams at PID7, the volume within the R13-treated injured rats was variably but significantly increased throughout the sensory/motor cortex, hippocampus, and thalamus as well as regions in the retrosplenial, agranular insular and piriform cortices, and in the striatum (Figure 4C, P<0.05).

**FIGURE 5:**
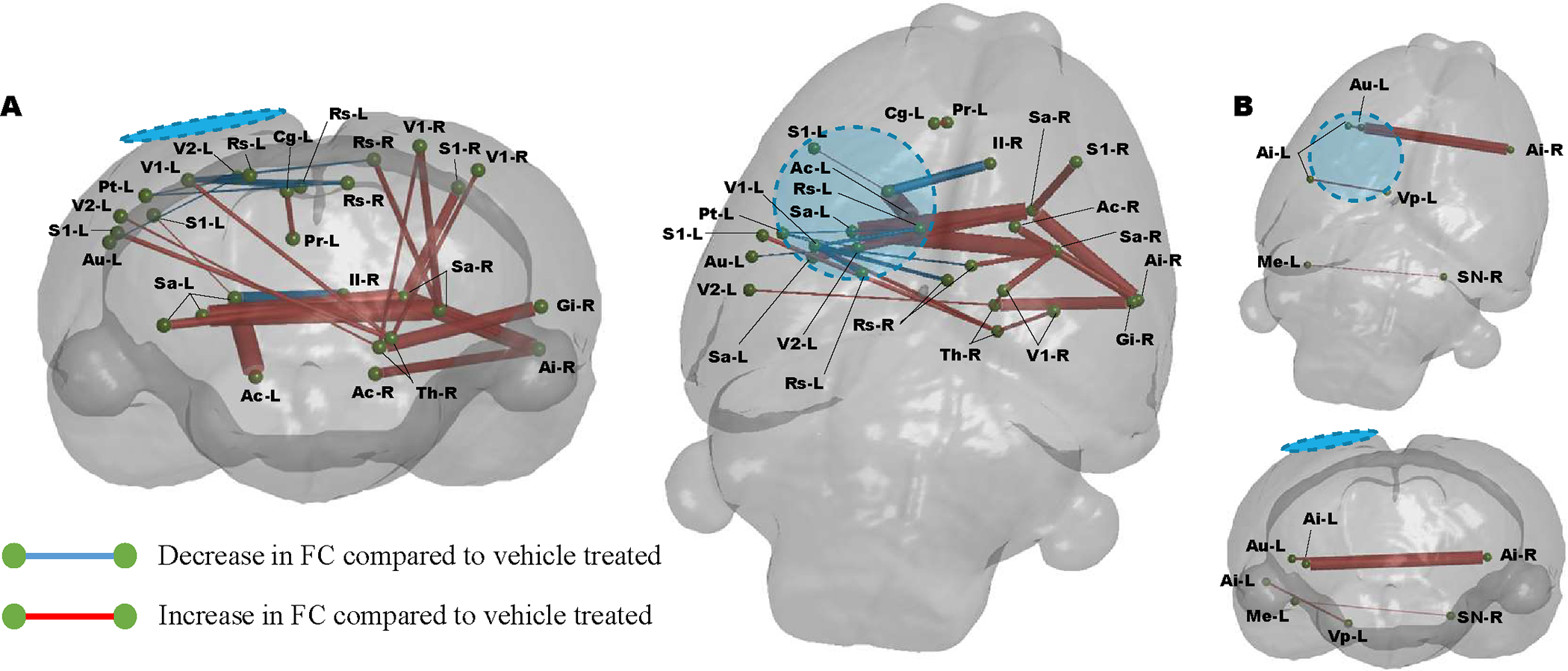
R13 treatment results in significant increases in FC at PID 7 compared to vehicle. Graphical coronal and planar plots showing the difference in functional connectivity over PIDs 1–7 that occurs due to R13 treatment compared to vehicle. R13 treatment results in significant increases in FC throughout the cortex compared to vehicle over the week of treatment (P<0.01, FDR corrected, N=8-R13, 10-Veh). **A)** In regions identified to have alter MD, **B)** In regions with MD similar to sham controls. Blue/hatched region indicate the approximate location of the craniectomy site. Key: Ac: Nucleus accumbens, Ai: Agranular insular cortex, Au: Auditory cortex, Cg: Cingulate cortex, Gi: Granular insular cortex, Il: Infralimbic cortex, Me: Medial entorhinal cortex, Pr: Perirhinal cortex, Pt: Parietal cortex, Rs: Retrospienial cortex, S1: Primary somatosensory cortex, Sa: Striatum, Sn: Substantia nigra, Th: Thalamus, V1: Primary visual cortex, V2: Secondary visual cortex, Vp: Ventral pallidum.

We next sought to determine whether or not the sustained or increased volumes of reduced MD after R13 treatment were associated with a worsened outcome, or whether it was an indication that R13-mediated metabolic support had acted favorably in some rats within the group to delay or prevent the inevitable tissue atrophy that is signaled by earlier pseudo-normalization of MD to sham levels, before rising above normal tissue values thereafter. We statistically compared the connectivity difference between vehicle and R13-treated, injured rats by computing the change in FC from PID1 to 7, and again limited the analysis to brain regions with low MD in injured rats at PID 1. We found that R13 significantly increased FC compared to vehicle-treated injured rats, and that this effect was centered upon transhemispheric connections at the cortical and subcortical level, and bilateral connections between cortex and subcortical regions (P<0.01, FDR corrected, Figure 5). Once again, we found very few significant effects of R13 on FC within regions of normal MD in comparison (Figure 5B). Importantly we tested for technical variance in the sham data by computing the change in FC over time, and found that no region-region difference over time survived FDR correction (data not shown).

**FIGURE 6:**
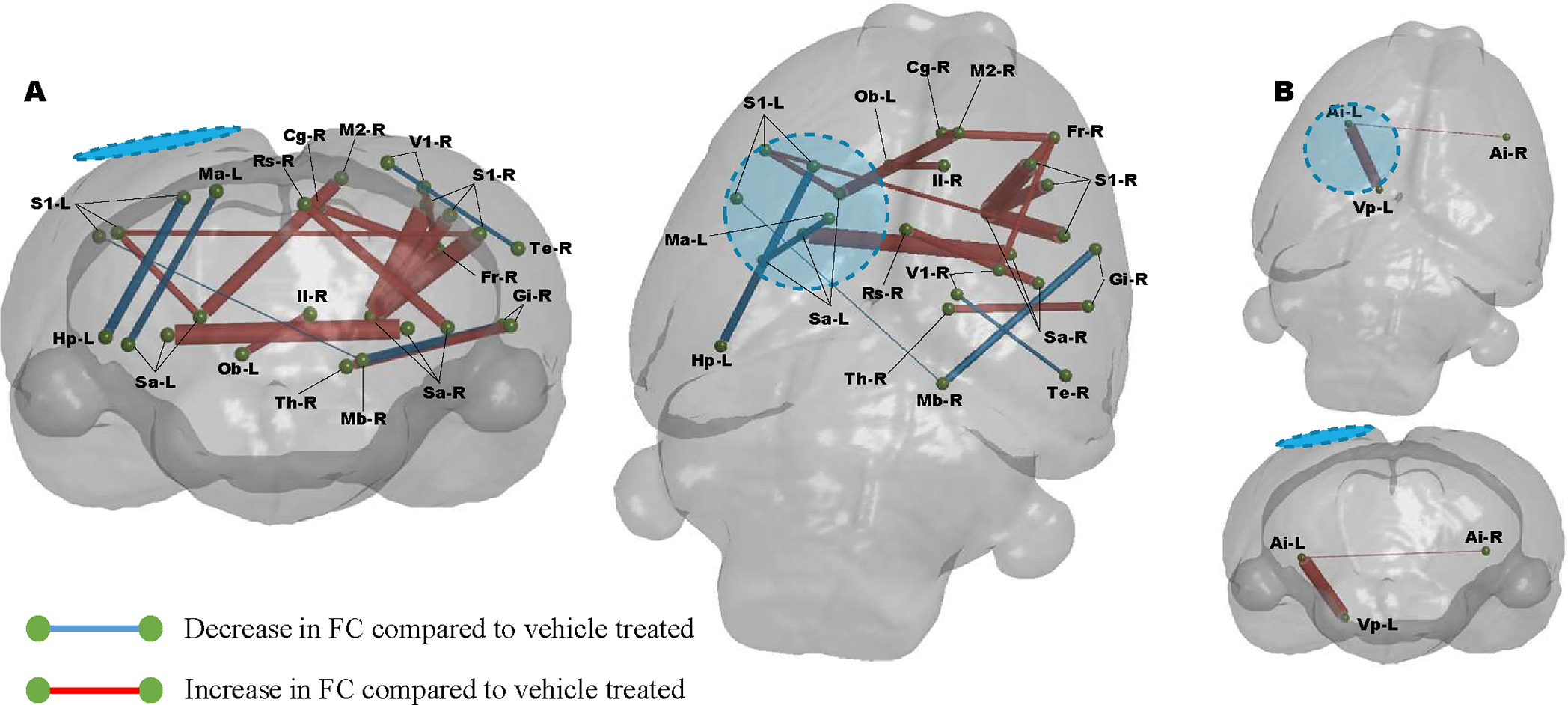
Injury-related disruption and drug effect on FC at 7 days post injury compared to vehicle treated rats. Graphical coronal and planar plots showing the difference in functional connectivity at PID 7. Daily R13 treatment from PID 1 resulted in significant increases in FC throughout the cortex compared to vehicle treated animals at PID 7. **A)** In regions identified to have alter MD, **B)** In regions with MD similar to sham controls. (P<0.01, FDR corrected, N=8-R13, 10-Veh. Blue/hatched region indicate the approximate location of the craniectomy site. Key: Ai: Agranular insular cortex, Cg: Cingulate cortex, Fr: Frontal cortex, Gi: Granular insular cortex, Hp: Hippocampus, Il: Infralimbic cortex, M2: Secondary motor cortex, Ma: Medial parietal association cortex, Mb: Rest of the midbrain, Ob: Olfactory bulb, Rs: Retrospienial cortex, S1: Primary somatosensory cortex, Sa: Striatum, Te: Temporal association cortex, Th: Thalamus, V1: Primary visual cortex, Vp: Ventral pallidum.

We confirmed the robustness of these data by conducting a cross-sectional comparison between both injured groups at PID 7 (Figure 6). While the results are similar to the temporal difference over the first week between the groups by way of striatal and contralateral cortex hyperconnectivity due to the effect of R13 (Figure 5), the comparison also showed a drug-induced recruitment of contralateral anterior cortical regions through increased contralateral connectivity from striatum to: cingulum, M2, frontal association and sensory cortex (Figure 6). Transhemispheric connectivity was also improved by R13 compared to vehicle through the sensory cortices, but ipsilateral sensory cortex to hippocampus was reduced.

**FIGURE 7:**
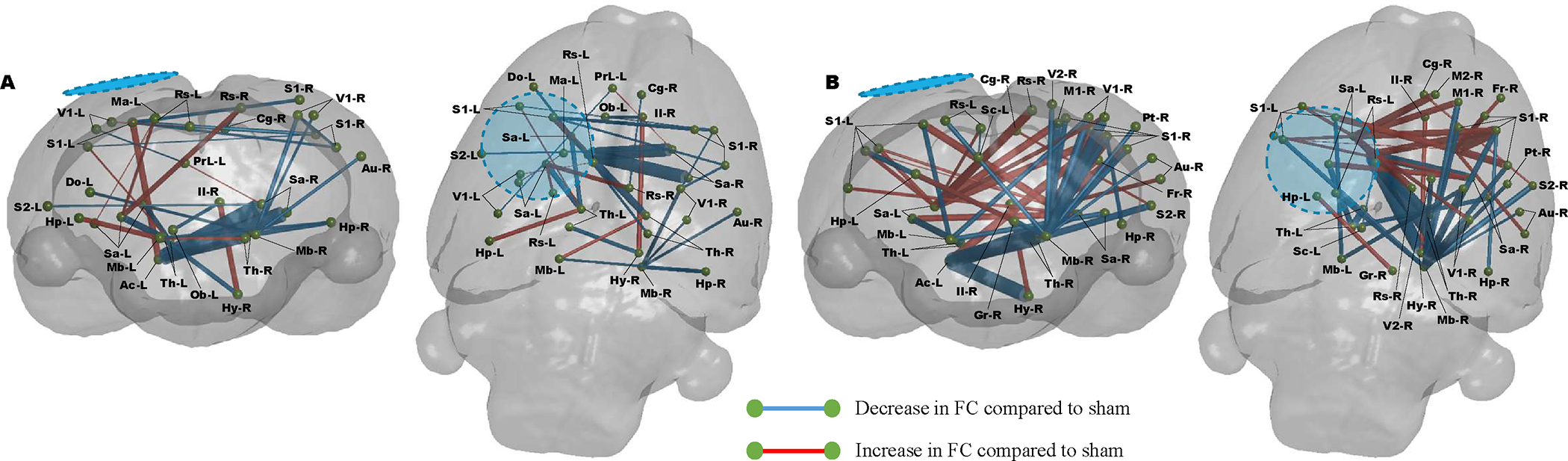
Injury-related disruption and drug effect on FC at 7 days post injury compared to sham rats. **A)** Injury with vehicle results in significant disruption in FC that remains altered from sham rats at PID 7, including several transhemispheric hypo- and hyper-connected regions. **B)** Daily R13 treatment from PID 1 resulted in significant increases in FC throughout the cortex compared to shams at PID 7, with mainly increases in cortico-thalamic and transhemispheric connectivity (P<0.01, FDR corrected, N=8-R13, 10-Veh, 10-Sham). Blue/hatched region indicate the approximate location of the craniectomy site. Key: Ac: Nucleus accumbens, Au: Auditory cortex, Cg: Cingulate cortex, Do: Dorsolateral orbital cortex, Fr: Frontal cortex, Gr: Central grey, Hp: Hippocampus, Hy: Hypothalamus, Il: Infralimbic cortex, M1: Primary motor cortex, M2: Secondary motor cortex, Ma: Medial parietal association cortex, Mb: Rest of the midbrain, Ob: Olfactory bulb, PrL: Prelimbic cortex, Pt: Parietal cortex, Rs: Retrospienial cortex, S1: Primary somatosensory cortex, S2: Secondary somatosensory cortex, Sa: Striatum, Sc: Superior colliculus, Th: Thalamus, V1: Primary visual cortex, V2: Secondary visual cortex.

Finally, we tested the degree of difference of each injured group from shams at 7 days (Figure 7). Surprisingly, connectivity within the vehicle-treated injured group had resolved closer to sham values, as assessed by the fewer connectivity differences globally compared to R13 (Figure 7a & 7b, respectively). The R13 treated group showed marked hyperconnectivity that emanated from around the ipsilateral primary cortical injury site and underlying striatum and thalamus across the brain to the contralateral hemisphere, as well as within the anterior part of the ipsilateral hemisphere. Of particular note was the marked reduction in FC from the contralateral midbrain to mostly posterior contralateral cortical regions, which, while present in the injured-vehicle group, was far less marked.

## 4. Discussion

These data show that injury-induced deficits in perfusion could be reasonably approximated by reductions in mean diffusivity (MD) acutely after injury, because a majority of regions with low perfusion matched to regions of low diffusivity, indicative of cell swelling. Regions of low MD were associated with reduced cross-brain functional connectivity (FC) and contralateral hyperconnectivity 1d after injury compared to sham, and also with altered network parameters indicative of disrupted local network topology. R13 intervention at 1–7d altered the tissue trajectory of MD pathology and resulted in a greater volume of tissue that remained with low MD at 7d. These regions were associated with a potentiation of cross-brain FC over 1–7d in R13 treated rats compared to vehicle, as well as increased FC within the contralateral hemisphere. Compared to sham at 7 days after injury, R13 enhanced ipsilateral and cross-hemisphere FC, but reduced midbrain to contralateral hemisphere FC.

### 4.1. Low MD as a Potential Acute Indicator of Metabolic Depression

Deficits in cerebral blood blow and metabolism after fluid percussion injury have been well documented [42, 43] similar to clinical TBI [44–49], and we show grossly similar results. We report that a high degree of spatial coincidence between deficits in CBF within a brain imaging slice and regional topography of low MD provides us with a measure to globally assess low MD as a proxy for altered flow-metabolism acutely after injury. Of course, a metabolic defect underlying low MD will have to be closely investigated in the same animal in future work using direct measures of mitochondrial function in order to more formally make use of it as an acute post-injury, indirect marker of altered metabolism. However, the evidence is strong for its use since decreases in MD have long been associated with cell swelling and altered perfusion after stroke [50, 51], and glycolytic and oxidative metabolic deficits are well known after FPI [52, 53]. We have also recently reported deficits in mitochondrial function at 3 days after FPI in data from our own lab [14]. The situation is of course far more complicated after FPI than stroke. The well-known hyperglycolysis that occurs early after TBI [53–55] may well mean that the CBF rate classically associated with membrane pump failure and associated cell swelling after stroke, is likely to be considerably higher after TBI. As a result, it’s not surprising that a CBF deficit of around 50% in this study is markedly associated with regions of low MD, indicating cellular swelling secondary to local metabolic depression.

### 4.2. Regions of Low MD are Associated with Altered Functional Connectivity

We found a consistent pattern of FC changes at 1d post-injury prior to drug intervention in which both increases and decreases of FC relative to sham, were predominantly associated with brain regions of low MD and not with regions of normal diffusivity. While we still do not know the exact structural and functional mechanism(s) underpinning changes in FC after injury, it is clear that they occur regionally coincident with metabolic and/or structural damage. We did not find a relationship between degree of MD reduction and the direction of FC change relative to sham. It will be important in future work to test for MD range as a biomarker of tissue trajectory; to use a range of injury severities to determine whether a greater MD reduction is associated with loss of FC, while a more mild or temporary reduction might be associated with hyperconnectivity.

The finding that R13-induced temporal increases in FC from 1–7d post-injury also occurred principally in these same regions of low MD may well reflect a metabolic effect of R13 on the underlying tissue. In support of this, we have shown in prior work that R13 significantly improves mitochondrial function at 3d after administration in the FPI model and at 7d using protein markers [14]. Further evidence of this metabolic effect comes from data herein where we measured a persistently increased volume of tissue with low MD due to drug at 7d compared to vehicle where MD returned to normal. To explain this effect, one must turn to the sequential tissue dynamics post-injury reported on by diffusion imaging. The usual trajectory of injured tissue with low MD is a pseudo-normalization to sham levels followed by increased MD indicative of cell death and a vasogenic environment, as classically seen after stroke [56]. The situation after FPI is similar [57, 58] with increased MD chronically [59]. The injured vehicle treated brains displayed the beginning of that temporal effect since the overlap between 1 and 7 of tissue with low MD was minor, indicating that MD levels had pseudo-normalized prior to presumed eventual tissue atrophy after 1–2 weeks post-injury.

However, the spatial overlap of low MD for R13-treated injured rats was much larger, and we postulate that this occurred because of metabolic support through R13 which offset cell membrane failure and prevented or at least delayed cell death. This is consistent with the reduction in the autophagy pathway cascade shown in data at 7d after FPI and R13 intervention [14]. The current data do support the idea that R13 was beneficial and not detrimental to brain function since it promoted increased connectivity above that of vehicle-treated injured rats, in agreement with a prior study improved cognitive outcome in the same model and drug administration regime [14]. Additionally, the prior study showed that R13 restored deficits GluR2, PSD-95, and p-CREB that were seen in TBI, indicating improved synaptic function through AMPA receptor activity [14]. Given the observed improvement in metabolic function and synaptic function previously reported with R13 treatment, alterations in FC were expected.

### 4.3. Hyperconnectivity of Subcortical and Contralateral Circuits Predominate after R13 Intervention

Early intervention with R13 resulted in clear changes in FC over that of vehicle treatment. These changes were principally centered upon increased FC between the hemispheres at the subcortical level and between the bilateral sensory areas, and within the contralateral hemisphere between striatum and thalamus to visual, sensory and retrosplenial cortex. These changes were replicated when a comparison to shams were made, but the extent of hypoconnectivity was shown to occur on a much greater scale than in vehicle treated injured rats. The success of R13 in reducing spatial memory deficits and anxiety-like behavior after FPI [14] is somewhat aligned with these changes, although determination of the specific circuits responsible cannot be achieved solely from these data.

However, the fact that R13 led to decreased ipsilateral hippocampal connectivity to sensory cortex suggest that the much larger subcortical increase in FC involving striatum and thalamus, with involvement of the retrosplenial cortex, a well-known hub region involved in memory formation [60] played an important role in the improvement in memory and anxiety-like behaviors. The heavy involvement of the striatum in the subcortical-to-cortical connectivity changes is surprising given its more anterior location away from the primary injury site. On the other hand, the biological relevance of striatal circuits to memory and anxiety-like behaviors cannot be overstated. The striatum is a crucial relay center from dorsal hippocampus for spatial memory [61, 62] and has long been associated with anxiety-like behavior [63, 64], so that enhanced FC from this region concurs with the R13-induced improvements in cognition and anxiety-like behavior shown before [14].

R13 induced increased transhemispheric connectivity at the level of sensory cortex but also through corticostriatal connections bilaterally to sensory and related cortical regions. This appropriation of contralateral circuits has been shown to occur spontaneously after contusion injury in rats [38]. One possibility is that it represents the path of least resistance to a way to coopt remote circuits to improve brain function. It remains to be seen whether this is transient post-injury, and whether it might persist chronically after early drug administration.

We observed a well-defined, antero-posterior separation of R13-induced changes in FC when comparing the data to shams at 7 days post-injury. There was a R13-induced posterior hypoconnectivity from mid-brain circuits to multiple posterior brain regions, while hyperconnectivity dominated the anterior aspect that. While these changes were present to a minor degree in injured vehicle treated brain, they were significantly less extreme. One possibility for the mid-brain hypoconnectivity is that it is a region further away from the surface coil used for MRI acquisition, so that low signal-to-noise (SNR) could play a role. However, comparing the SNR across this region in the brain revealed that there was no significant difference to all other regions (data not shown). It therefore remains likely that R13 promoted changes in FC remote from the injury site, an effect also seen after contusion injury [38].

In summary, the majority of LFPI-induced changes in functional connectivity occurred in regions of cell swelling and/or metabolic deficits. The effect of the BDNF mimetic R13 was to alter connectivity in the direction of sham controls, and in predominantly metabolically compromised regions rather than in unaffected regions. This therefore indicates a likely metabolic effect of the drug acutely after injury.

## Highlights

- Mild FPI is associated with significant but transient reductions in rCBF.
- Regions of low MD are associated with altered functional network architecture.
- R13 intervention altered the temporal trajectory of tissue with low MD.
- Hyperconnectivity of subcortical and contralateral circuits predominate with R13.
